# Improved In Vitro Anti-*Mucorales* Activity and Cytotoxicity of Amphotericin B with a Pegylated Surfactant

**DOI:** 10.3390/jof8020121

**Published:** 2022-01-27

**Authors:** Kévin Brunet, Cheikh A. B. Diop, Alexia Chauzy, Noémie Prébonnaud, Sandrine Marchand, Blandine Rammaert, Frédéric Tewes

**Affiliations:** 1 INSERM U1070, Pôle Biologie Santé, 1 rue Georges Bonnet, 86022 Poitiers, France; bambandao19@gmail.com (C.A.B.D.); alexia.chauzy@univ-poitiers.fr (A.C.); noemie.prebonnaud@etu.univ-poitiers.fr (N.P.); sandrine.marchand@univ-poitiers.fr (S.M.); blandine.rammaert.paltrie@univ-poitiers.fr (B.R.); frederic.tewes@univ-poitiers.fr (F.T.); 2 Faculté de Médecine et Pharmacie, Université de Poitiers, 6 rue de la Milétrie, 86073 Poitiers, France; 3 Service de Mycologie-Parasitologie, Département des Agents Infectieux, CHU de Poitiers, 2 rue de la Milétrie, 86021 Poitiers, France; 4 Service de Toxicologie-Pharmacocinétique, CHU de Poitiers, 2 rue de la Milétrie, 86021 Poitiers, France; 5 Service de Maladies Infectieuses et Tropicales, CHU de Poitiers, 2 rue de la Milétrie, 86021 Poitiers, France

**Keywords:** *Mucorales*, antifungal combination, amphotericin B

## Abstract

The aim of this study was to evaluate the effect of the combination of amphotericin B (AmB) and various non-ionic surfactants on the anti-*Mucorales* activity of AmB, the toxicity of the combination on eukaryotic cells and the modification of AmB aggregation states. Checkerboards were performed on five genera of *Mucorales* (12 strains) using several combinations of different surfactants and AmB. These data were analyzed by an E_max_ model. The effect of surfactants on the cytotoxic activity of AmB was then evaluated for red blood cells and two eukaryotic cell lines by absorbance and propidium iodide internalization. Finally, the effect of polyethylene glycol (15)-hydroxystearate (PEG15HS) on the aggregation states of AmB was evaluated by UV-visible spectrometry. PEG15HS increased the efficacy of AmB on four of the five *Mucorales* genera, and MICs of AmB were decreased up to 68-fold for *L. ramosa*. PEG15HS was the only surfactant to not increase the cytotoxic activity of AmB. Finally, the analysis of AmB aggregation states showed that the increased efficacy of AmB and the absence of toxicity are related to an increase in monomeric and polyaggregated forms of AmB at the detriment of the dimeric form. In conclusion, PEG15HS increases the in vitro efficacy of AmB against *Mucorales* at low concentration, without increasing its toxicity; this combination could therefore be evaluated in the treatment of mucormycosis.

## 1. Introduction

Mucormycosis is a life-threatening invasive fungal disease caused by a species belonging to *Mucorales* order [1]. They are difficult-to-treat infections in immunocompromised patients, being resistant to most antifungal drugs [2]. Actual treatment is based, whenever possible, on surgery, control of underlying diseases, and aggressive antifungal therapy [3,4]. Currently, there are only three active antifungal agents against *Mucorales*: Amphotericin B formulated as liposomes (L-AmB), recommended for first-line therapy [3], and posaconazole and isavuconazole, as alternative options [3,5]. Despite treatment, mortality remains inacceptable, varying from 30 to 90%, according to the disease forms [2].

In this context, combinations of antifungal agents have been tested in vitro and in vivo to improve their efficacy against *Mucorales*. Unfortunately, no combination of antifungal agents has been shown to be more effective than an antifungal alone against mucormycosis in patients [6,7,8,9,10,11]. Several authors have therefore studied combinations of antifungal agents and non-antifungal molecules [12]. Some of these combinations increased antifungal efficiency in vitro or in vivo in animal models, but also presented issues such as improving *Mucorales* virulence, toxicity or lack of approval by the medical regulation authorities [11]. Hence, the search for anti-*Mucorales* associations based on AmB, the most powerful antifungal agent currently known against *Mucorales*, must be further developed to improve the treatment of mucormycosis.

Several mechanisms of antifungal action of AmB have been proposed [13]. Most of them involve the interaction of AmB with ergosterol, the main sterol found in fungal membranes. The most described mechanism is the formation of a barrel-shaped ion channel due to the assembly of AmB and ergosterol within the plasma membrane, which induces leakage of ions through the fungal plasma membrane. It has also been shown that AmB can kill fungi by simply binding ergosterol, without the need to create the membrane ions channel [14].

Since the affinity of AmB for ergosterol is greater than that for cholesterol, the sterol present in mammalian cell membranes, its toxicity is selective against fungi at relatively low concentrations [15]. However, at high concentrations, AmB also binds to cholesterol and produces a toxic effect. AmB binds to sterols because of its structure and amphiphilic property. Its property also allows AmB to organize in different aggregation states, mainly monomeric, dimeric (self-aggregate, aggregated) or poly-aggregated (super-aggregated) forms. The relative proportion of these aggregated forms depends on the nature of the medium in which it is dissolved and on its concentration [16]. Several authors have shown that the aggregation states of AmB influence its efficacy and toxicity because they have different selective affinities for ergosterol and cholesterol [17]. The dimeric form is the most toxic, and the super-aggregated form is the least toxic [16,17].

Therefore, controlling the state of aggregation of AmB and promoting the ploy-aggregated form through its association with amphiphilic molecules such as surfactants could increase its efficacy and decrease its toxicity. Nonionic surfactants are the most used to prepare parenteral formulations because they are the least toxic and have the lowest critical micellar concentration (CMC), thus easily forming stable micelles [18]. They are generally less hemolytic and less irritating, and they tend to maintain near physiological pH values when in solution compared to other types of surfactants [19]. Thus, the aim of this study was to evaluate the in vitro effect of different nonionic surfactants, which have already been used to develop parenteral formulations, on the anti-*Mucorales* efficacy of AmB and on its cytotoxicity against mammalian cells, in connection with its state of aggregation.

## 2. Materials and Methods

### 2.1. Strains

Twelve clinical isolates of Mucorales identified by ARNr sequencing were used: Rhizopus arrhizus (formerly oryzae) (5), Rhizopus microsporus (1), Lichtheimia corymbifera (3), Lichtheimia ramosa (1), Mucor circinelloides (1), and Rhizomucor pusillus (1). Isolates were subcultured from frozen stocks (−80 °C) on 75 cm^3^ flasks containing 15 mL of Sabouraud dextrose agar (Sigma-Aldrich, St. Quentin Fallavier, France) over 3 days at 37 °C. Spores were collected by flooding the flask with 10 mL of sterile water. The suspensions were filtered on nylon filter (Millipore, Carrigtwohill, Ireland) with pore size of 11μm to remove hyphal elements. The concentration of the spore suspensions was then adjusted in sterile water to obtain inocula of 2 × 10^5^ spores/mL.

### 2.2. Media

RPMI 1640 (with L-glutamine, pH indicator, no bicarbonate) (Sigma-Aldrich, Saint-Quentin-Fallavier, France) supplemented with 2% (*w*/*v*) dextrose and buffered to pH 7 with 0.165 mol/L MOPS (Sigma-Aldrich) was used for minimum inhibitory concentration (MIC) and hemolytic activity measurements. This medium was sterilized using a 0.22 µm pore-size filter. Sterile Dulbelcco’s phosphate buffered saline (DDPBS, Dominique Dutscher, Bernolsheim, France) of pH 7 was also used as a medium to measure the hemolytic activity of AmB.

### 2.3. Solutions

**Figure 1:**
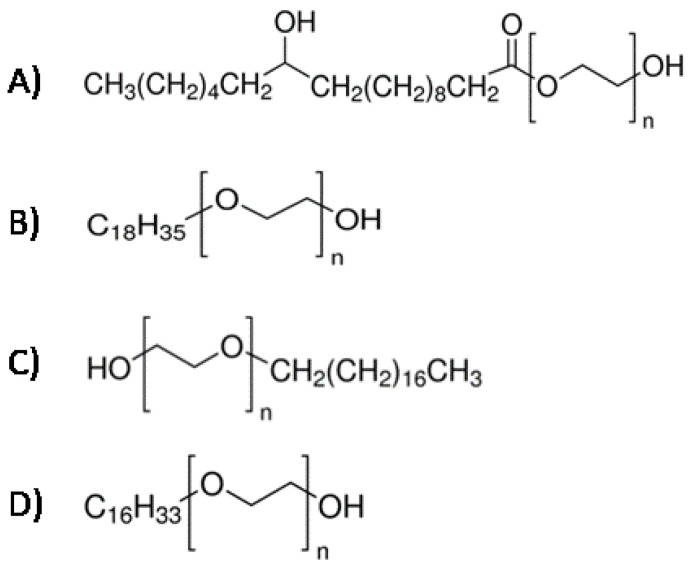
Structure of the nonionic surfactants tested. (**A**) PEG15HS with *n* = 15; (**B**) Brij^®^ O20 with *n* = 20; (**C**) Brij^®^ S10 with *n* = 10, or Brij^®^ S20 if *n* = 20; (**D**) Brij^®^ C10 with *n* = 10.

AmB powder (Sigma-Aldrich) was dissolved in dimethyl sulfoxide to obtain a stock solution at 8192 mg/L, stored at −80 °C for six months. On the day of use, AmB and freshly made surfactant solutions were diluted in the same medium (RPMI or DPBS) to obtain the desired concentrations. Various surfactants were used (Figure 1), a polyethyleneglycol fatty alcohol ester (polyethylene glycol (15)-hydroxystearate, PEG15HS, named Solutol^®^ HS15, Kolliphor^®^ HS 15, or Macrogol 15 Hydroxystearate) and different polyethyleneglycol fatty ethers having variable hydrophobic and hydrophilic parts (polyoxyethylene (10) stearyl ether (Brij^®^ S10); polyoxyethylene (20) stearyl ether (Brij^®^ S20); polyoxyethylene (20) oleyl ether (Brij^®^ O20); and polyoxyethylene (10) cetyl ether (Brij^®^ C10)).

### 2.4. Checkerboard Assays

The AmB MIC of each strain of *Mucorales* was measured in the presence of different concentrations of PEG15HS, as described by the European Committee on Antimicrobial Susceptibility Testing (EUCAST) broth microdilution antifungal susceptibility test methodology, with modification for the checkerboard procedure [20]. The AmB MIC of *L. ramosa* (the strain of *Mucorales* that responded the most to the AmB-PEG15HS combination) was also measured with other nonionic polyethoxylated surfactants (Brij^®^ surfactants).

Checkerboards were performed by adding 100 µL of two-fold dilutions of AmB (0.008 to 4 mg/L) and surfactant solutions (0.04 to 1024 mg/L) in 96-well plates. One hundred microliters of a spore suspension at a final concentration of 10^5^ spores/mL was added to the wells, and the plates were incubated for 24 h at 37 °C. MICs were read visually after 24 h as the lowest concentrations of AmB that completely inhibited the growth of the fungal strain. Three experiments were performed for each strain.

### 2.5. Checkerboard Data Modeling

An inhibitory E_max_ model (Equation (1)), proposed by Chauzy et al. [21] to evaluate the enhancing effect of non-antibiotic molecules on the efficacy of antibiotics against bacteria, was used here to describe the decrease in AmB MIC values (MIC_surf_) against various *Mucorales* strains in relation to surfactant concentrations (C_surf_).

MIC_surf_ = MIC_0_ − ((MIC_0_ − MIC_∞_) × C_surf_)/(EC_50_ + C_surf_) (1)

In Equation (1), MIC_0_ refers to the AmB MIC measured without surfactants and MIC_∞_ is the asymptotic value of AmB MIC when the surfactant concentration (C_surf_) tends toward infinity. The maximal ratio (R_max_) in AmB MIC decrease, defined as maximal efficacy, was calculated as the ratio of MIC_0_/MIC_∞._ EC_50_ is the concentration of surfactant producing 50% of R_max_ and characterizes the potency of the surfactants. These parameters were determined using WinNonlin software (version 6.2, Certara, NJ, USA) as described by Chauzy et al. [21].

### 2.6. Hemolytic Activity

The hemolytic activity of AmB in the presence of surfactants was measured according to the protocols previously described [22]. This measure was performed on two media: DPBS, classically used in the literature for hemolytic activity tests and allowing us to compare our results to other studies, and RPMI, used for antifungal activity tests. Human blood samples from different healthy volunteers were collected and centrifuged at 3000× *g* for 5 min. Plasma was removed and the erythrocytes were washed four times with 2 mL of 0.9% NaCl by centrifugation at 3000× *g* for 5 min. Erythrocytes were suspended in RMPI or DPBS, and their concentration was adjusted to 5 × 10^8^ cells/mL. First, erythrocytes were incubated with different combinations of AmB (0.5 mg/L) and surfactants (0, 5, 10, 20, 50, 100 mg/L) for 1 h at 37 °C under shaking, then centrifuged at 3000× *g* for 5 min. Then, 100 μL of the supernatant was transferred in a flat-bottomed transparent 96-well plate (SARSTEDT, France), and the absorbance was measured at 540 nm using a plate reader (Infinite M200 PRO, Tecan^®^, France). Untreated erythrocytes dispersed in RPMI or DPBS were used as a negative control, and erythrocytes treated by 1% (*v*/*v*) of TritonX-100 were used as a positive control. The percentage of hemolysis was calculated using the following equation: 100 × (A_sample_ − A_negative_)/(A_positive_ − A_negative_), where A_sample_ is the absorbance measured for each experimental condition, and A_negative_ and A_positive_ are the absorbance of negative and positive controls, respectively. Experiments were performed in duplicate. After these preliminary experiments, experiments were repeated with combinations of AmB (0, 1, 5, 15 mg/L) and PEG15HS (0, 5, 10, 20, 50, 100, 200, 400 mg/L) in DPBS and combinations of AmB (0, 15, 30, 45 and 60 mg/L) and PEG15HS (0, 5, 10, 20, 50, 100, 200, 400 mg/L) in RPMI. The experiments were performed in duplicate on four experiments for RPMI and DPBS.

### 2.7. Cytotoxicity

The effect of surfactants on the cytotoxicity of AmB was evaluated on two types of human cell lines: human adenocarcinoma cells (A549) cultivated as an adherent monolayer, and human monocytic cells (THP-1) cultivated in suspension in culture medium. Cells were used between passages 21 and 24 for A549 cells and passages 5 and 6 for THP-1 cells. Cells were cultured in RPMI supplemented with 10% *v/v* of fetal calf serum and 17.5 mg/L of β-mercaptoethanol and incubated at 37 °C under 90–95% RH and 5% *v/v* of CO_2_ in the air. [23]. For the experiments, THP-1 cells were seeded in 96-well plates at 5.10^4^ cells/well, and A549 cells were used at 90% of confluence. Cells were washed with DPBS and incubated with different concentrations of AmB (4 and 30 mg/L) and PEG15HS (0, 5, 10, 20, 50, 100, 200, 400 mg/L) in the culture medium. Toxicity was assessed by evaluating the cellular internalization of propidium iodide (PI). Indeed, PI penetrates into cells and fluoresces only if the plasma membrane is damaged [24]. Its uptake was measured by measuring its fluorescence (excitation λ = 560 nm, emission λ = 630 nm), which was recorded every 2 min for 10 min using an Infinite M200 Pro plate reader (Tecan, France). PI uptake was calculated as follows: PI uptake (%) = (F_obs_ − F_0_)/(F_100_ − F_0_) × 100, where F_obs_ was the fluorescence measured for a given PEG15HS concentration in the presence of AmB (4 or 30 mg/L), F_0_ was the fluorescence observed in the absence of PEG15HS or AmB, and F_100_ was the fluorescence measured in the presence of Triton 0.1% (*v/v*). Both experiments were performed in duplicate.

### 2.8. Determination of AmB Aggregation States

The determination of AmB aggregation states was performed by UV-visible spectrometry as described previously [25]. Indeed, the aggregation state of AmB can be assessed using UV-visible spectroscopy [16,17] because the absorbance of the heptaene group of AmB is very sensitive to conformational changes such as those induced during aggregation. Hence, the monomeric form of AmB is characterized by a well-defined spectrum having an absorbance maximum at 408–410 nm. When AmB is aggregated, the spectrum is blue-shifted, and two new maxima appear, one at 338 nm, attributed to the dimeric form of AmB, and the other at 330 nm, attributed to the poly-aggregated form [25,26]. With these two maxima being relatively close to each other, a broad intermediate peak is often observed, mainly when the two forms coexist. Briefly, mixtures of 100 µL of AmB (1, 2, 4, 15, 30, 45, 60 mg/L) and 100 µL of PEG15HS (1, 5, 10, 15, 20, 30, 50, 60, 100, 200, 400 mg/L) were transferred to 96-well plates in RPMI and DPBS. Absorbance spectra between 300 and 420 nm were recorded, with a resolution of 2 nm, using a plate reader (Infinite M200 PRO, Tecan). Absorbance for the blank solution was subtracted, and the ratios of the absorbance of the monomeric form (maximum at 410 nm) to that of the dimeric form (maximum at 338 nm) were assessed for each combination of PEG15HS and AmB.

### 2.9. Statistics

Statistical analysis was performed using Prism 8 software (Graph Pad, La Jolla, CA, USA). Quantitative variables were expressed as mean and standard deviation, and means were compared with Kruskall-Wallis and Mann-Whitney tests.

## 3. Results

### 3.1. Nonionic Surfactants Enhanced AmB Activity against Various Mucorales Isolates

**Figure 2:**
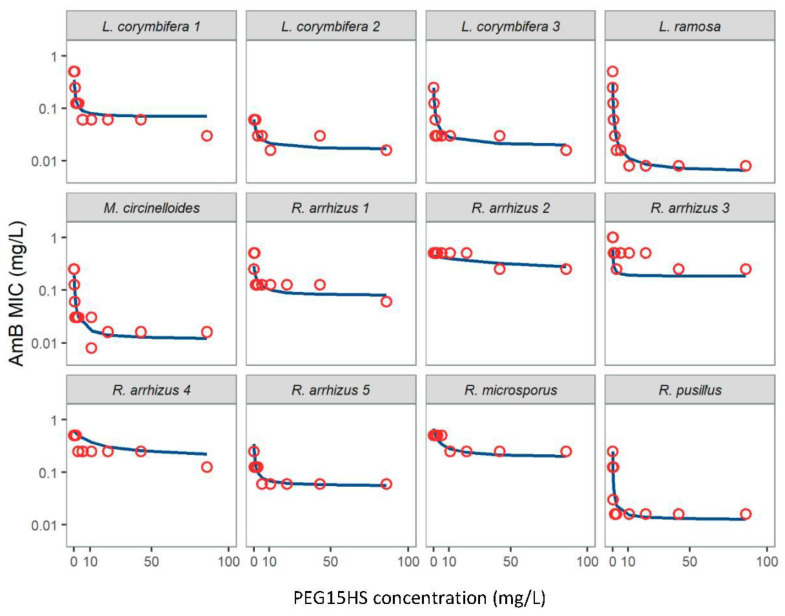
Amphotericin B (AmB) MICs (mg/L) versus polyethylene glycol (15)-hydroxystearate (PEG15HS) concentrations (mg/L) for twelve *Mucorales* strains. Circles represent the AmB MICs determined during one checkerboard experiment, and the solid lines the typical AmB MIC profiles predicted by the E_max_ model based on three checkerboard experiments.

The first step of the study was to evaluate the ability of PEG15HS to improve the efficacy of AmB against several genera and strains of *Mucorales* involved in mucormycosis (Figure 2).

All isolates had an MIC for AmB alone less than 1 mg/L, while MIC values greater than 1024 mg/L were obtained for PEG15HS alone (Table 1). The decrease in the MIC of AmB as a function of PEG15HS concentrations was adequately described by the inhibitory E_max_ model (Figure 2). The maximum efficacy (R_max_) and potency (EC_50_) of surfactants to decrease the MIC of AmB against the different strains were used as comparison variables (Table 1).

The R_max_ of PEG15HS to improve the antifungal action of AmB varied from 2.5 to 63.8, depending on the isolate (Table 1). The highest value was obtained for the species *L. ramosa*. R_max_ values were also relatively high against *L. corymbifera* species, expect for one strain (*L. corymbifera* 2), but the efficacy of AmB alone against this strain was already high, as shown by its low MIC_0_ value of 0.06 mg/L. Thus, it seems difficult to further reduce the already low MIC of AmB against this strain. The combination AmB-PEG15HS was also effective against the tested strains of *R. pusillus* and *M. circinelloides*, with an R_max_ of 20 and 16.5, respectively. However, for the two species of the genus *Rhizopus* tested (one *R. microsporus* and five strains of *R. arrhizus*), the combination seemed less effective, with R_max_ values below 4, with the exception of one strain of *R. arrhizus*, which had an R_max_ value of 6.4 (Table 1).

When R_max_ values were above 4, PEG15HS was generally very potent, with EC_50_ values below 0.5 mg/L, showing that PEG15HS improved AmB efficacy at low concentrations. When R_max_ was less than 4, as for most of *Rhizopus* strains, EC_50_ values were mainly above 1 mg/L, and could increase up to 53.45 mg/L.

To assess whether the effect was specific for PEG15HS or could also be obtained with other nonionic polyethoxylated surfactants, the antifungal efficacy of AmB associated with different Brij^®^ was also tested against *L. ramosa*, the strain of *Mucorales* that responded the most to the AmB-PEG15HS combination. The Brij^®^ surfactants tested are structurally related to PEG15HS, with a PEG group composed of 10 to 20 ethylene oxide units and a different lipophilic part, i.e., a cetyl chain for Brij^®^ C10 and a oleyl chain for Brij^®^ O20, or a related lipophilic part, i.e., a stearyl chain for Brij^®^ S10 and Brij^®^ S20.

**Figure 3:**
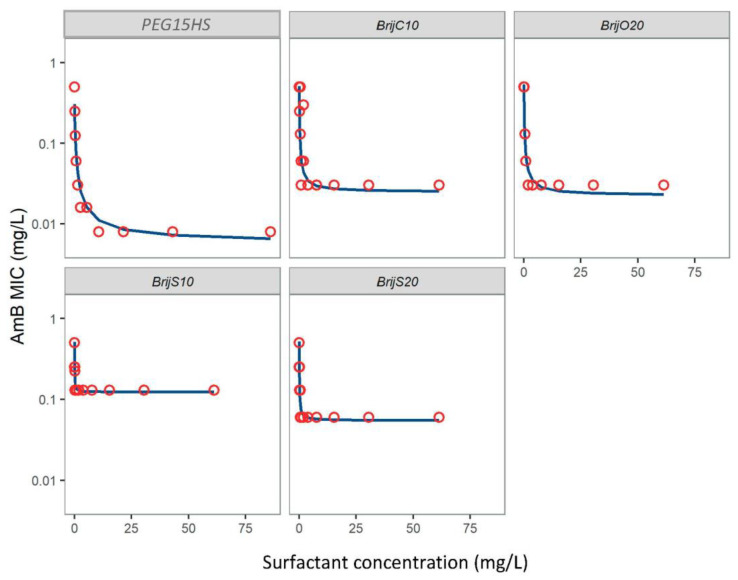
MIC of AmB against *Lichtheimia ramosa*, measured for different concentrations of surfactants. Circles represent the AmB MICs determined during one checkerboard experiment, and the solid lines the typical AmB MICs predicted by the E_max_ model, based on three checkerboard experiments.

Brij^®^ S10, Brij^®^ S20, Brij^®^ 020 and Brij^®^ C10 were also able to increase the efficacy of AmB on *L. ramosa* (Figure 3). The four nonionic surfactants tested showed high potencies, with EC_50_ values below 0.1 mg/L (Table 1). However, while Brij^®^ 020 and Brij^®^ C10 presented efficacy close to that of PEG15HS (23.2 and 20.4, respectively), Brij^®^ S10 and Brij^®^ S20 were less efficient, with an efficacy of 4.1 and 9.2, respectively (Table 1).

### 3.2. The Effect of AmB on the Integrity of Mammalian Cell Membranes Was Not Potentiated by PEG15HS at the Concentration Producing the Maximum Effect

The results presented above show that certain nonionic polyethoxylated surfactants, mainly PEG15HS, Brij^®^ 020 and Brij^®^ C10, were able to potentiate the antifungal activity of AmB against various isolates of *Mucorales*. However, these results are only interesting if the potential human cytotoxicity of AmB is not increased and the combinations have favorable benefit/risk ratios. The potentialization of AmB cytotoxicity by surfactants was therefore evaluated.

**Figure 4:**
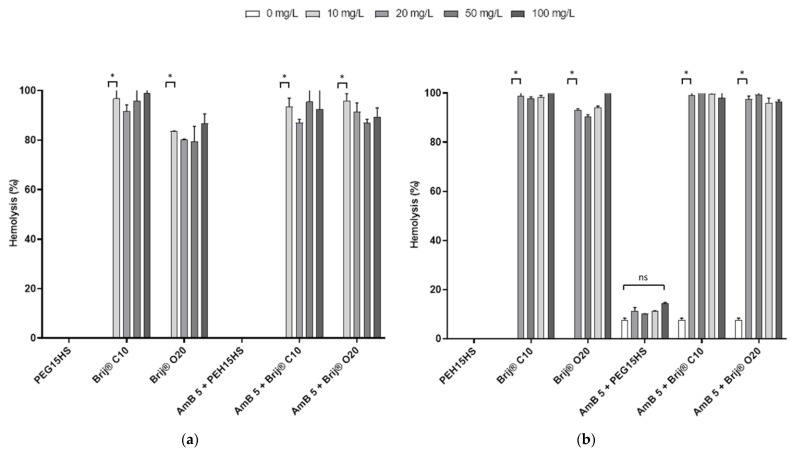
Hemolytic activity of AmB, surfactants and combinations of AMB and surfactants. Hemolytic activity of PEG15HS, Brij^®^ 020 and Brij^®^ C10 (0, 10, 20, 50 and 100 mg/L), AmB (5 mg/L) and combinations of AmB (5 mg/L) and PEG15HS, Brij^®^ 020 or Brij^®^ C10 (0, 10, 20, 50 and 100 mg/L) was measured on human erythrocytes dispersed in RPMI (**a**) or in DPBS (**b**) after 1 h of incubation at 37 °C. Hemolysis was measured at 540 nm using a plate reader. Experiments were performed in duplicate. ns: non-significant; * *p* < 0.05.

To evaluate this potential adverse effect, the hemolytic activity of 5 mg/L of AmB alone or in the presence of various concentrations of PEG15HS, Brij^®^ O20 and Brij^®^ C10 (0, 10, 20, 50 and 100 mg/L) was first measured on human erythrocytes dispersed in RPMI (Figure 4a) or in DPBS (Figure 4b). Interestingly, PEG15HS alone was not hemolytic in the concentration range tested in both media, while Brij^®^ O20 and Brij^®^ C10 alone were both highly hemolytic (greater than 90% hemolysis) from the lowest concentration tested (10 mg/L) in both media. At 5 mg/L, AmB alone was not hemolytic in RPMI (Figure 4a), whereas it hemolyzed 7.7 ± 0.7% of erythrocytes when they were dispersed in DPBS (Figure 4b). PEG15HS at concentrations up to 100 mg/L did not potentiate the hemolytic activity of AmB in both media (Figure 4).

**Figure 5:**
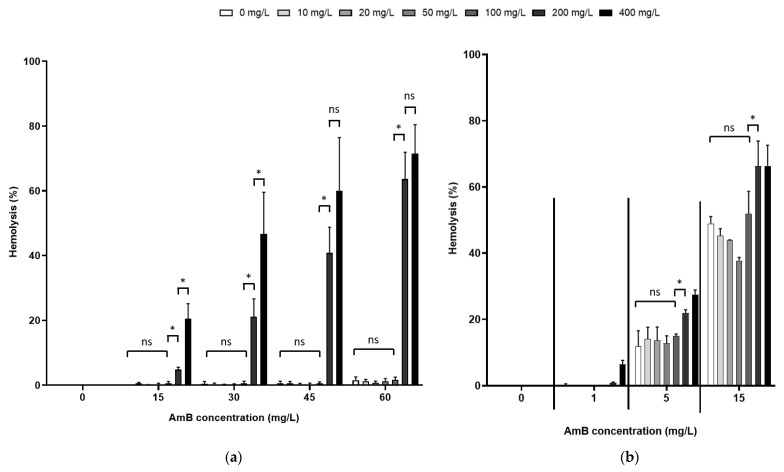
Hemolytic activity of AmB, polyethylene glycol (15)-hydroxystearate (PEG15HS) and combinations of AmB and PEG15HS. Hemolytic activity of AmB (0, 1, 5, 15, 30, 45 and 60 mg/L), PEG15HS (0, 10, 20, 50, 100, 200 and 400 mg/L) and combinations of AmB (0, 1, 5, 15, 30, 45 and 60 mg/L) and PEG15HS (0, 10, 20, 50, 100, 200 and 400 mg/L) was measured on human erythrocytes dispersed in RPMI (**a**) and in DPBS (**b**) after 1 h of incubation at 37 °C. Hemolysis was measured at 540 nm using a plate reader. Experiments were performed in duplicate on two experiments. ns: non-significant; * *p* < 0.05.

Brij^®^ 020 and Brij^®^ C10 were highly hemolytic at the concentrations tested; studies with these two surfactants were discontinued, and additional cytotoxicity studies were performed only with PEG15HS. Further hemolytic experiments were performed with different concentrations of AmB (1, 5, 15, 30, 45 and 60 mg/L) and PEG15HS (0, 10, 20, 50, 100, 200 and 400 mg/L) (Figure 5). For concentrations up to 100 mg/L, PEG15HS did not increase the hemolytic activity of AmB, regardless of the medium and AmB concentrations. However, for PEG15HS concentrations of 200 and 400 mg/L, AmB hemolytic activity increased in both media. This increase in hemolytic activity was more notable in RPMI than in DPBS. For example, for 15 mg/L of AmB, the hemolysis was 0.6 ± 0.6% in RPMI for 100 mg/L of PEG15HS and increased to 20.5 ± 4.7% for 400 mg/L of PEG15HS. In DPBS, the hemolysis was 52.0 ± 6.7% in RPMI for 100 mg/L of PEG15HS and increased to 66.4 ± 6.3% for 400 mg/L of PEG15HS. Again, the hemolytic activity of AmB was much higher in DPBS than in RPMI. For example, for 15 mg/L pure AmB, 49.0 ± 2.1% hemolysis was observed in DPBS, while less than 0.1% was obtained in RPMI at this AmB concentration.

**Figure 6:**
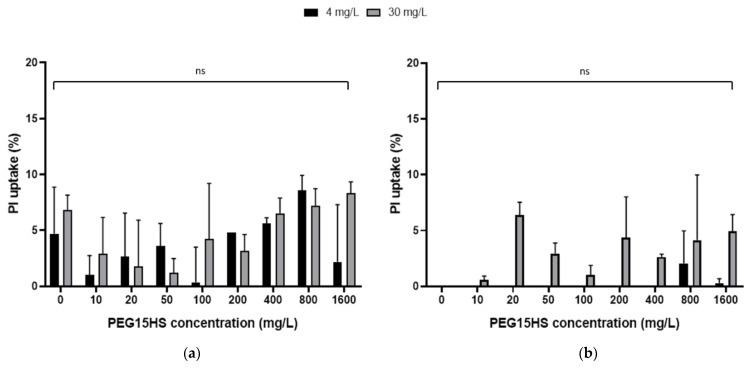
Propidium iodide uptake of A549 alveolar epithelial cells and THP-1 monocytes incubated with combinations of AmB and PEG15HS. THP-1 (**a**) and A549 (**b**) cell lines were incubated with AmB alone (4 and 30 mg/L) and AmB–PEG15HS combinations (0, 10, 20, 50, 100, 200, 400, 800 and 1600 mg/L). Propidium iodide (PI) uptake was evaluated by fluorescence measurement (excitation λ = 560 nm, emission λ = 630 nm) every 2 min for 10 min. Experiments were performed in duplicate. ns: non-significant.

Additional testing of plasma membrane integrity in the presence of AmB–PEG15HS combinations was performed on other cell types similar to those found in the lungs: the adherent cell line A549, representing alveolar epithelial cells, and the THP-1 cell line, representing mononuclear immune cells (monocytes). The membrane integrity of these cells was evaluated by measuring the cellular uptake of PI (Figure 6).

At concentrations of 4 and 30 mg/L, AmB incubated for one hour with cells induced a PI uptake of 4.7 ± 4.2% and 6.8 ± 1.3%, respectively, in THP-1 (Figure 6a) and 0% in A549 cells (Figure 6b). The addition of PEG15HS at a concentration up to 1600 mg/L did not significantly potentiate this uptake in either cell line (Figure 6).

### 3.3. PEG15HS Alters Aggregation States of AmB

Since previous results have shown that PEG15HS increases the activity of AmB against *Mucorales* without increasing its toxicity, a spectrometric study was performed to understand if these results were linked to a modulation of AmB aggregation states.

**Figure 7:**
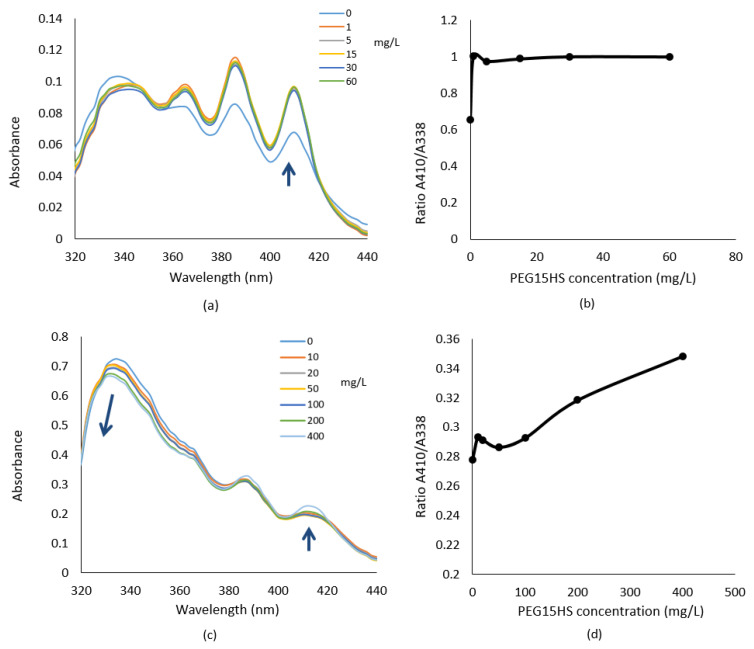
Absorbance spectra of AmB in presence of PEG15HS. Absorbance spectra of mixtures of 100 µL of AmB at 4 mg/L (**a**) or at 15 mg/L (**c**) and 100 µL of PEG15HS (1, 5, 10, 15, 20, 30, 50, 60, 100, 200 and 400 mg/L) in RPMI. The ratios of the absorbance of the monomeric form (410 nm) to that of the dimeric form (338 nm) were assessed for each concentration of PEG15HS and AmB at 4 mg/L (**b**) or at 15 mg/L (**d**). Three experiments were performed in duplicate, and one experiment was represented. Each arrow shows a spectra shift, a major increase or decrease of absorbance.

Figure 7a shows the absorption spectra of AmB (at 4 mg/L) in RPMI in the presence of increasing PEG15HS concentrations (0–60 mg/L). This AmB concentration is the lowest concentration that allowed accurate spectra to be recorded. At this concentration, two maximums of relatively similar intensity were present: one at 410 nm, showing the presence of monomer, and one at 338 nm, specific to the dimeric form [27]. The addition of PEG15HS produced an increase in the peak at 410 nm and a red shift of the peak at 338 nm, associated with a decrease in its intensity. Spectral modifications induced by aggregation of AmB can be represented by the ratio of A410/A338 absorbances (Figure 7b). The higher the ratio, the more monomeric AmB was detected. The ratio of the monomeric form to the dimeric form was rapidly increased upon addition of PEG15HS (Figure 7b).

For a high concentration of AmB (15 mg/L), AmB was found in the dimeric form, as indicated by a high absorbance at 330–350 nm (Figure 7c). PEG15HS at 100 mg/L led to a slight decrease of the dimeric form but did not increase the monomeric form. Adding PEG15HS above 100 mg/L resulted in a decrease of the dimeric form and an increase of the monomeric form (Figure 7c,d). Moreover, adding PEG15HS to 15 mg/L AmB increased the formation of polyaggregated form, as shown by the blue shift of the peak at 338 nm (Figure 7c).

## 4. Discussion

Mucormycosis is an emerging problem in infectious disease due to the low efficacy of antifungal agents. AmB is the first-line treatment, but mortality remains unacceptably high, ranging from 30 to 90%, depending on the form of the disease (rhinocerebral, pulmonary, disseminated) [9]. It is therefore important to search for effective combinations based on AmB, currently the most active drug against *Mucorales*. Since the aggregation states of AmB influence its activity and toxicity, and surfactants have the capacity to modify these aggregation states [16], the combination of AmB with surfactants approved by health agencies (FDA, EMA) for parenteral use has been evaluated as a means of improving its efficacy while limiting its toxicity.

An inhibitory E_max_ model was used to describe the checkerboard results. Pharmacodynamic interactions between anti-infectious agents used in combination are generally studied by performing checkerboard experiments to determine the fractional inhibitory concentration index (FIC index) [21,28]. This index is based on the comparison between the MIC of each molecule alone and the MIC of the combination. This index is used to determine the characteristics of an association: synergistic (FIC index ≤ 0.5), additive (>0.5 and ≤4) or antagonistic (>4). However, this index cannot be calculated when one of the molecules has no anti-infective effect and no MIC value. The E_max_ model was used in this context to describe the interaction between an antifungal drug (AmB) and non-antifungal compounds (non-ionic surfactants), whereas the FIC index cannot be used to assess the efficacy of these combinations in this condition. This model has already been used to evaluate antifungal combinations against *Candida auris* [29] and for β-lactam and β-lactamase inhibitor combinations against bacteria [21], but to our knowledge not for antifungal/non-antifungal combinations. The use of this model enables evaluation of the potency (EC_50_) and of the efficacy of adjuvant (R_max_), to select the most promising combination for PK/PD testing [21].

Our results showed that the PEG15HS improves the efficacy of AmB against various *Mucorales*. Indeed, the PEG15HS–AmB combination had an R_max_ between 2 and 64 against various *Mucorales* responsible for mucormycosis. These results are difficult to compare with the combinations reported in the literature, since the FIC index is generally used. To obtain a synergistic effect based on the FIC index, the MIC of the two compounds must be divided by at least four. Thus, an R_max_ ≥ 4 can be considered representative of a synergic association. However, the E_max_ model offers more precision than the FIC index to determine the strength of a synergy. Interestingly, most of the molecules tested in the literature in combination with AmB have an antifungal activity even if they are not antifungal drugs. In our study, PEG15HS showed no antifungal activity up to concentrations of 1024 mg/L. This type of molecule, without antifungal activity, should not allow the selection of resistant fungi [30], which reinforces the interest in this association.

PEG15HS is a non-ionic surfactant, listed as an inactive ingredient in the FDA database. It is used as a solubilizer in marketed oral, parenteral and ophthalmic formulations [31,32]. It has low toxicity and excellent biocompatibility due to low hemolytic properties [33,34,35,36] and thus represents a potentially interesting synergistic agent with AmB. Other tested surfactants also improved the efficacy of AmB against *L. ramosa*, suggesting that the effect of non-ionic surfactants on the antifungal activity of AmB seems to be due to a class effect of surfactants, of which PEG15HS is the most effective among those tested.

Efficacy against *Mucorales* was variable according to genus, with a lower efficacy ratio predicted against the genus *Rhizopus*. This low efficacy of PEG15HS to improve the antifungal activity of AmB could be attributed to a relatively low proportion of ergosterol, which is the target of AmB in the membrane of this genus of *Mucorales*. Ergosterol is the most predominant sterol in fungi and is generally present in more than 50% and often up to 85–96% of total sterols [37]. However, studies have shown that ergosterol is the main sterol in most *Mucorales* [37,38,39], including *Rhizopus arrhizus* [40]. Interestingly, Dannaoui et al. showed that rifampicin was synergistic with AmB on 100% of the strains of *Lichtheimia* sp., while it was synergistic on only 40% of the strains of *Rhizopus* sp. [41]. The authors did not provide a hypothesis for this species-specific effect, but it appears that *Rhizpous* sp. seems to be less susceptible to AmB-based combinations.

The PEG15HS-AmB combination was also effective against *Aspergillus fumigatus* (data not shown). AmB is the first-line treatment for azole-resistant aspergillosis, but it is associated with higher mortality compared to treatment with azoles due to lower efficacy [42]. Hence, the use of the PEG15HS–AmB combination could be interesting in the treatment of azole-resistant *Aspergillus.* We also performed some tests on *Candida albicans* and *C. glabrata*, which showed that PEG15HS decreases the MIC of AmB against these two species (data not shown). These results suggest that this combination could be used in several types of fungal infections, but further studies are needed.

One of the limitations of a drug combination is the potential increase in toxicity compared to drugs alone. AmB is known to be toxic to erythrocytes and eukaryotic cells due to the formation of pores in their plasma membranes by interaction with cholesterol and the induction of oxidative reactions [43]. All surfactants tested were hemolytic at the concentrations tested (10 to 100 mg/L), except for PEG15HS. Indeed, our results showed that, up to 100 mg/L, PEG15HS did not potentiate the hemolytic activity of AmB. This is consistent with the fact that PEG15HS is used in human parenteral preparations at concentrations above 100 mg/L [31]. Moreover, PEG15HS has been used in parenteral preparations for anticancer chemotherapy on mice at concentrations greater than 100 mg/L, without toxicity [35].

Interestingly, the hemolytic activity of AmB was much higher in DPBS than in RPMI. In 1983, Meyer et al. showed that AmB toxicity on erythrocytes is linked to the ionic strength and the ionic composition of the media [44]. They noticed more alterations of the erythrocyte membrane in medium with reduced ionic strength. Several authors have shown that the monomer/dimer ratio of AmB decreases with increasing K^+^ concentration [25]. Since the aggregation states of AMB are linked to its toxicity, differences in ion concentration in RPMI or DPBS could modify the monomer/dimer ratio in these media and change the hemolytic activity of AmB.

The amplifying effect of the antifungal activity of AmB was found with all five surfactants tested on different genera of *Mucorales* and other genera of fungi. Thus, it appears that this effect is not surfactant or fungus specific. UV-visible spectra strongly suggested that a modification of AmB aggregation states is induced by PEG15HS. The aggregation states of AmB are linked to various factors, such as its concentration, pH, temperature, ionic strength and the presence of excipients, especially surfactants in the medium [16]. Our results suggested that PEG15HS increased the efficacy of AmB by increasing the proportion in monomer of AmB. This result is consistent with other studies that showed that due to its high affinity for ergosterol, the monomeric form is the most active [16,17]. These data are in agreement with the study of Tancrède et al., which showed that two surfactants, one nonionic (lauryl sucrose) and one anionic (sodium deoxycholate), enhanced AmB selectivity for ergosterol at the concentration that induced monomerization of AmB [45]. They suggested that below the CMC of the surfactant (in the absence of micelles), surfactant molecules penetrated and destroyed AmB aggregates, releasing the monomeric form [45]. A further increase in surfactant concentration led to a micellar structure composed of surfactant molecules incorporated with AmB monomers. Furthermore, they showed that the CMC of the nonionic surfactant was altered by the presence of AmB, while that of the anionic surfactant was not, and they deduced that AmB interacted more strongly with the nonionic surfactant than with the anionic one. The CMC of PEG15HS is 90 mg/L [33], and the increase in AmB efficacy occurred at a PEG15HS concentration less than 10 mg/L, thus in the absence of micelles. Thus, the increase in antifungal efficacy of AmB could also be due to the degradation of AmB aggregates by PEG15HS monomers, releasing AmB monomers, which are more effective than dimers against fungi.

Adding PEG15HS to 15 mg/L AmB slightly increases the proportion of monomers and increase the formation of polyaggregated form, as shown by our results. Data from the literature indicate that the polyaggregated forms are less toxic in vivo and in vitro than the other forms [16]. Indeed, the dimeric form of AmB was the most toxic in mice, followed by the monomeric form, whereas polyaggregated forms have the lowest toxicity. Authors have shown that the aggregation state of deoxycholate AmB coincides with the dimeric form. When deoxycholate AmB was heated for 1 h at 70 °C, it increased the monomeric and polyaggregated forms of AmB, resulting in lower toxicity in mice [16]. Similarly, Barwicz et al. [17] showed that AmB combined with non-ionic surfactant (lauryl sucrose) has a less acute toxicity in vivo in mice than fungizone due to the monomerization of AmB. Several studies have shown that AmB polyaggregates have a low affinity for ergosterol and cholesterol and act as a reservoir for the monomeric form [16,46,47,48]. The lack of increase in AmB toxicity in the presence of PEG15HS could therefore be explained by the decrease of the dimeric form and the increase of the polyaggregated form. However, the absolute monomeric concentration of AmB increased with the PEG15HS concentration and thus can reach a toxic concentration, which is higher than for the dimeric form. Interestingly, the increase of the AmB hemolytic activity in RPMI and DPBS was observed for a PEG15HS concentration just higher than the CMC (90 mg/L). The low toxicity of commercial liposomal amphotericin B is also related to the presence of AmB in polyaggregated form, but also to the slow release of AmB from the liposomes [16]. However, while liposomal amphotericin B has a lower toxicity than deoxycholate AmB, its activity against fungi is generally not increased against *Mucorales* and other fungi.

To conclude, PEG15HS enhanced the AmB activity against *Mucorales* with high efficacy and potency. Due to the low toxicity of PEG15HS, its high potency, and the need for low concentrations, the AmB–PEG15HS combination could improve the treatment of mucormycosis.

## Figures and Tables

**Table 1:** Parameter estimates of the inhibitory E_max_ model for the strains of *Mucorales* in the presence of polyethylene glycol (15)-hydroxystearate (PEG15HS) or different Brij^®^ surfactants.

Surfactant	Isolates	MIC_0_ (mg/L)	EC_50_ (mg/L)	MIC_∞_ (mg/L)	R_max_ (MIC0/MIC_∞_)
PEG15HS	*L. corymbifera* 1	0.36	0.44	0.068	5.2
	*L. corymbifera* 2	0.06	1.33	0.016	3.8
	*L. corymbifera* 3	0.25	0.42	0.019	13.0
	*L. ramosa*	0.51	0.19	0.008	63.8
	*M. circinelloides*	0.19	0.32	0.012	16.5
	*R. arrhizus* 1	0.28	1.34	0.077	3.7
	*R. arrhizus* 2	0.44	53.45	0.177	2.5
	*R. arrhizus* 3	0.59	0.28	0.183	3.2
	*R. arrhizus* 4	0.61	8.57	0.186	3.3
	*R. arrhizus* 5	0.35	0.47	0.055	6.4
	*R. microsporus*	0.68	2.32	0.190	3.6
	*R. pusillus*	0.25	0.13	0.012	19.9
Brij^®^ S10	*L. ramosa*	0.51	0.02	0.123	4.1
Brij^®^ S20	*L. ramosa*	0.51	0.02	0.056	9.2
Brij^®^ O20	*L. ramosa*	0.51	0.06	0.022	23.2
Brij^®^ C10	*L. ramosa*	0.51	0.05	0.025	20.4

MIC_0_: MIC of AmB in the absence of surfactant. EC_50_: surfactant concentration producing 50% of R_max_. MIC_∞_: asymptotic value of AmB MIC when surfactant concentration tends toward infinity.

## Data Availability

Not applicable.

## References

[1] Farmakiotis D., Kontoyiannis D.P. (2016). Mucormycoses. Infect. Dis. Clin. North Am..

[2] Serris A., Danion F., Lanternier F. (2019). Disease Entities in Mucormycosis. J. Fungi.

[3] Cornely O.A., Alastruey-Izquierdo A., Arenz D., Chen S.C.A., Dannaoui E., Hochhegger B., Hoenigl M., Jensen H.E., Lagrou K., Lewis R.E. (2019). Global Guideline for the Diagnosis and Management of Mucormycosis: An Initiative of the European Confederation of Medical Mycology in Cooperation with the Mycoses Study Group Education and Research Consortium. Lancet Infect. Dis..

[4] Tissot F., Agrawal S., Pagano L., Petrikkos G., Groll A.H., Skiada A., Lass-Flörl C., Calandra T., Viscoli C., Herbrecht R. (2017). ECIL-6 Guidelines for the Treatment of Invasive Candidiasis, Aspergillosis and Mucormycosis in Leukemia and Hematopoietic Stem Cell Transplant Patients. Haematologica.

[5] Sipsas N.V., Gamaletsou M.N., Anastasopoulou A., Kontoyiannis D.P. (2018). Therapy of Mucormycosis. J. Fungi.

[6] Abidi M.Z., Sohail M.R., Cummins N., Wilhelm M., Wengenack N., Brumble L., Shah H., Jane Hata D., McCullough A., Wendel A. (2014). Stability in the Cumulative Incidence, Severity and Mortality of 101 Cases of Invasive Mucormycosis in High-Risk Patients from 1995 to 2011: A Comparison of Eras Immediately before and after the Availability of Voriconazole and Echinocandin-Amphotericin Combination Therapies. Mycoses.

[7] Kyvernitakis A., Torres H.A., Jiang Y., Chamilos G., Lewis R.E., Kontoyiannis D.P. (2016). Initial Use of Combination Treatment Does Not Impact Survival of 106 Patients with Haematologic Malignancies and Mucormycosis: A Propensity Score Analysis. Clin. Microbiol. Infect..

[8] van Burik J.-A.H., Hare R.S., Solomon H.F., Corrado M.L., Kontoyiannis D.P. (2006). Posaconazole Is Effective as Salvage Therapy in Zygomycosis: A Retrospective Summary of 91 Cases. Clin. Infect. Dis..

[9] Lanternier F., Dannaoui E., Morizot G., Elie C., Garcia-Hermoso D., Huerre M., Bitar D., Dromer F., Lortholary O., French Mycosis Study Group (2012). A Global Analysis of Mucormycosis in France: The RetroZygo Study (2005–2007). Clin. Infect. Dis..

[10] Reed C., Bryant R., Ibrahim A.S., Edwards J., Filler S.G., Goldberg R., Spellberg B. (2008). Combination Polyene-Caspofungin Treatment of Rhino-Orbital-Cerebral Mucormycosis. Clin. Infect. Dis..

[11] Brunet K., Rammaert B. (2020). Mucormycosis Treatment: Recommendations, Latest Advances, and Perspectives. J. Mycol. Med..

[12] Schwarz P., Cornely O.A., Dannaoui E. (2019). Antifungal Combinations in Mucorales: A Microbiological Perspective. Mycoses.

[13] Kamiński D.M. (2014). Recent Progress in the Study of the Interactions of Amphotericin B with Cholesterol and Ergosterol in Lipid Environments. Eur. Biophys. J..

[14] Gray K.C., Palacios D.S., Dailey I., Endo M.M., Uno B.E., Wilcock B.C., Burke M.D. (2012). Amphotericin Primarily Kills Yeast by Simply Binding Ergosterol. Proc. Natl. Acad. Sci. USA.

[15] Adler-Moore J.P., Gangneux J.-P., Pappas P.G. (2016). Comparison between Liposomal Formulations of Amphotericin B. Med. Mycol..

[16] Espada R., Valdespina S., Alfonso C., Rivas G., Ballesteros M.P., Torrado J.J. (2008). Effect of Aggregation State on the Toxicity of Different Amphotericin B Preparations. Int. J. Pharm..

[17] Barwicz J., Christian S., Gruda I. (1992). Effects of the Aggregation State of Amphotericin B on Its Toxicity to Mice. Antimicrob. Agents Chemother..

[18] Tewes F., Corrigan O.I., Healy A.M. (2015). Surfactants in Pharmaceutical Products and Systems. Encyclopedia of Pharmaceutical Science and Technology.

[19] Jiao J. (2008). Polyoxyethylated Nonionic Surfactants and Their Applications in Topical Ocular Drug Delivery. Adv. Drug Deliv. Rev..

[20] European Committee on Antimicrobial Susceptibility Testing EUCAST DEFINITIVE DOCUMENT E.DEF 9.3.2. https://www.eucast.org/fileadmin/src/media/PDFs/EUCAST_files/AFST/Files/EUCAST_E_Def_9.3.2_Mould_testing_definitive_revised_2020.pdf.

[21] Chauzy A., Buyck J., de Jonge B.L.M., Marchand S., Grégoire N., Couet W. (2019). Pharmacodynamic Modelling of β-Lactam/β-Lactamase Inhibitor Checkerboard Data: Illustration with Aztreonam-Avibactam. Clin. Microbiol. Infect..

[22] Serrano D.R., Hernández L., Fleire L., González-Alvarez I., Montoya A., Ballesteros M.P., Dea-Ayuela M.A., Miró G., Bolás-Fernández F., Torrado J.J. (2013). Hemolytic and Pharmacokinetic Studies of Liposomal and Particulate Amphotericin B Formulations. Int. J. Pharm..

[23] Zhang D., Wang L., Wang H., Lv X., Ren Q., Zheng G. (2021). The Effects of Legumain in THP1 Leukemia Cells. Biocell.

[24] Lankoff A., Sandberg W.J., Wegierek-Ciuk A., Lisowska H., Refsnes M., Sartowska B., Schwarze P.E., Męczyńska-Wielgosz S., Wojewódzka M., Kruszewski M. (2012). The Effect of Agglomeration State of Silver and Titanium Dioxide Nanoparticles on Cellular Response of HepG2, A549 and THP-1 Cells. Toxicol. Lett..

[25] Gagoś M., Arczewska M. (2011). Influence of K^+^ and Na^+^ Ions on the Aggregation Processes of Antibiotic Amphotericin B: Electronic Absorption and FTIR Spectroscopic Studies. J. Phys. Chem. B.

[26] Gruda I., Dussault N. (1988). Effect of the Aggregation State of Amphotericin B on Its Interaction with Ergosterol. Biochem. Cell Biol..

[27] Gagoś M., Hereć M., Arczewska M., Czernel G., Dalla Serra M., Gruszecki W.I. (2008). Anomalously High Aggregation Level of the Polyene Antibiotic Amphotericin B in Acidic Medium: Implications for the Biological Action. Biophys. Chem..

[28] White R.L., Burgess D.S., Manduru M., Bosso J.A. (1996). Comparison of Three Different in Vitro Methods of Detecting Synergy: Time-Kill, Checkerboard, and E Test. Antimicrob. Agents Chemother..

[29] Bidaud A.L., Botterel F., Chowdhary A., Dannaoui E. (2019). In Vitro Antifungal Combination of Flucytosine with Amphotericin B, Voriconazole, or Micafungin against Candida Auris Shows No Antagonism. Antimicrob. Agents Chemother..

[30] Schneider E.K., Reyes-Ortega F., Velkov T., Li J. (2017). Antibiotic-Non-Antibiotic Combinations for Combating Extremely Drug-Resistant Gram-Negative “Superbugs”. Essays Biochem..

[31] Shaukat Ali Kolliphor® HS 15—An Enabler for Parenteral and Oral Formulations. http://www.americanpharmaceuticalreview.com/Featured-Articles/358749-Kolliphor-HS-15-An-Enabler-for-Parenteral-and-Oral-Formulations/.

[32] U.S. Food and Drug Administration Inactive Ingredient Search for Approved Drug Products. https://www.accessdata.fda.gov/scripts/cder/iig/index.cfm.

[33] Younes N.F., Abdel-Halim S.A., Elassasy A.I. (2018). Solutol HS15 Based Binary Mixed Micelles with Penetration Enhancers for Augmented Corneal Delivery of Sertaconazole Nitrate: Optimization, in Vitro, Ex Vivo and in Vivo Characterization. Drug Deliv..

[34] Hou Y., Zhang F., Lan J., Sun F., Li J., Li M., Song K., Wu X. (2019). Ultra-Small Micelles Based on Polyoxyl 15 Hydroxystearate for Ocular Delivery of Myricetin: Optimization, in Vitro, and in Vivo Evaluation. Drug Deliv..

[35] Yan H., Zhang Z., Jia X., Song J. (2016). D-α-Tocopheryl Polyethylene Glycol Succinate/Solutol HS 15 Mixed Micelles for the Delivery of Baohuoside I against Non-Small-Cell Lung Cancer: Optimization and in Vitro, in Vivo Evaluation. Int. J. Nanomed..

[36] Coon J.S., Knudson W., Clodfelter K., Lu B., Weinstein R.S. (1991). Solutol HS 15, Nontoxic Polyoxyethylene Esters of 12-Hydroxystearic Acid, Reverses Multidrug Resistance. Cancer Res..

[37] Weete J.D., Gandhi S.R. (1997). Sterols of the Phylum Zygomycota: Phylogenetic Implications. Lipids.

[38] Weete J.D., Abril M., Blackwell M. (2010). Phylogenetic Distribution of Fungal Sterols. PLoS ONE.

[39] Müller C., Neugebauer T., Zill P., Lass-Flörl C., Bracher F., Binder U. (2018). Sterol Composition of Clinically Relevant Mucorales and Changes Resulting from Posaconazole Treatment. Molecules.

[40] Weete J.D., Lawler G.C., Laseter J.L. (1973). Total Lipid and Sterol Components of Rhizopus Arrhizus: Identification and Metabolism. Arch. Biochem. Biophys..

[41] Dannaoui E., Afeltra J., Meis J.F.G.M., Verweij P.E., Eurofung Network (2002). In Vitro Susceptibilities of Zygomycetes to Combinations of Antimicrobial Agents. Antimicrob. Agents Chemother..

[42] van der Linden J.W.M., Snelders E., Kampinga G.A., Rijnders B.J.A., Mattsson E., Debets-Ossenkopp Y.J., Kuijper E.J., Van Tiel F.H., Melchers W.J.G., Verweij P.E. (2011). Clinical Implications of Azole Resistance in Aspergillus Fumigatus, The Netherlands, 2007–2009. Emerg. Infect. Dis..

[43] Hamill R.J. (2013). Amphotericin B Formulations: A Comparative Review of Efficacy and Toxicity. Drugs.

[44] Meyer H.W., Richter W., Winkelmann H. (1983). Nystatin- and Amphotericin B-Induced Structural Alterations of the Erythrocyte Membrane: Importance of Reduced Ionic Strength. Exp. Pathol..

[45] Tancrède P., Barwicz J., Jutras S., Gruda I. (1990). The Effect of Surfactants on the Aggregation State of Amphotericin B. Biochim. Biophys. Acta.

[46] Huang W., Zhang Z., Han X., Tang J., Wang J., Dong S., Wang E. (2002). Ion Channel Behavior of Amphotericin B in Sterol-Free and Cholesterol- or Ergosterol-Containing Supported Phosphatidylcholine Bilayer Model Membranes Investigated by Electrochemistry and Spectroscopy. Biophys. J..

[47] Golenser J., Domb A. (2006). New Formulations and Derivatives of Amphotericin B for Treatment of Leishmaniasis. Mini Rev. Med. Chem..

[48] Chéron M., Petit C., Bolard J., Gaboriau F. (2003). Heat-Induced Reformulation of Amphotericin B-Deoxycholate Favours Drug Uptake by the Macrophage-like Cell Line J774. J. Antimicrob. Chemother..

